# Supplementary material for: Identification of Genomic Regions for Traits Associated with Flowering in Cassava (Manihot esculenta Crantz)
Source: Plants (Basel). 2024 Mar 12;13(6):796. doi: 10.3390/plants13060796 (PMC10974989; doi:10.3390/plants13060796)
Supplement: Supplementary file 1 [file plants-13-00796-s001.zip › Plants_Supplementaries2/Supplementary Figure 1.pdf]

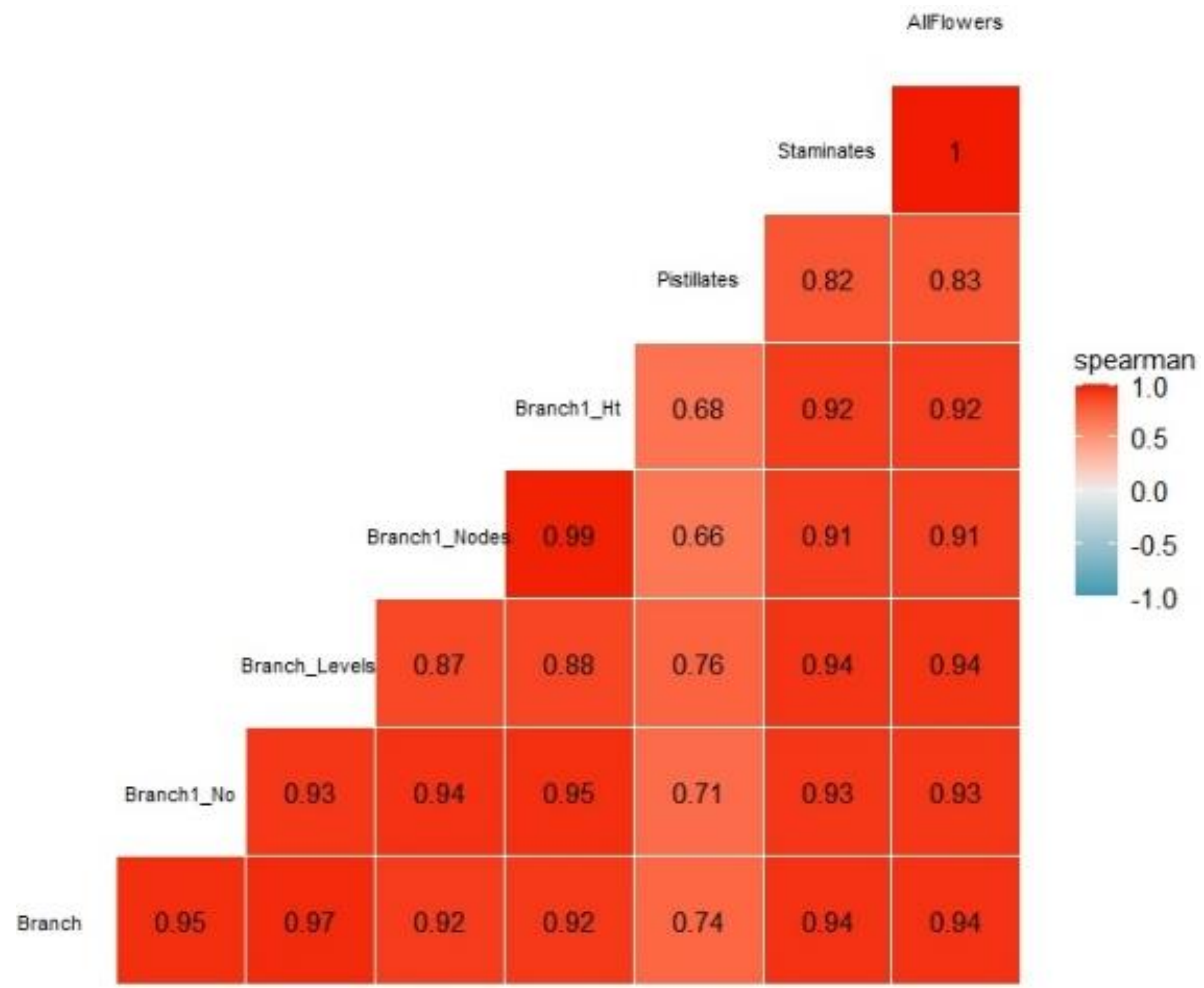

Supplementary Figure 1: Spearman correlation coefficients (r) for flowering-associated traits and flower types evaluated across two seasons and locations. Branch1_No = branch type at first branching; Branch1_Nodes = number of nodes at first branching; Branch1_Ht = stem height at first branching
