# Supplementary material for: Identification of Genomic Regions for Traits Associated with Flowering in Cassava (Manihot esculenta Crantz)
Source: Plants (Basel). 2024 Mar 12;13(6):796. doi: 10.3390/plants13060796 (PMC10974989; doi:10.3390/plants13060796)
Supplement: Supplementary file 1 [file plants-13-00796-s001.zip › Plants_Supplementaries2/Supplementary Table 1_Clustered accessions with parents.pdf]

Female	Male	Accession	Cluster
UG15F088P005	UG15F271P008	UG16F002P001	1
UG15F088P005	UG15F271P008	UG16F002P004	1
UG15F196P020	UG15F088P006	UG16F004P004	1
UG15F088P005	UG15F088P005	UG16F006P001	1
UG15F116P007	UG15F168P009	UG16F059P001	1
UG15F088P005	UG15F088P005	UG16F060P001	1
UG15F117P006	UG15F196P007	UG16F061P003	1
UG15F088P005	UG15F119P001	UG16F062P001	1
UG15F258P004	UG15F203P005	UG16F063P002	1
UG15F168P001	UG15F203P005	UG16F065P002	1
UG15F127P004	UG15F127P004	UG16F066P001	1
UG15F088P006	UG15F117P012	UG16F074P004	1
UG15F276P001	UG15F271P008	UG16F075P004	1
UG15F306P011	UG15F047P002	UG16F076P001	1
UG15F088P005	UG15F185P004	UG16F077P001	1
UG15F168P001	UG15F117P012	UG16F078P001	1
UG15F258P004	UG15F306P034	UG16F083P003	1
UG15F168P001	UG15F118P008	UG16F084P001	1
UG15F193P010	UG15F235P007	UG16F098P001	1
UG15F088P003	UG15F258P004	UG16F100P002	1
UG15F088P002	UG15F118P008	UG16F109P001	1
UG15F251P008	UG15F088P009	UG16F115P001	1
UG15F088P006	UG15F258P004	UG16F117P001	1
UG15F088P009	UG15F203P005	UG16F127P005	1
UG15F185P004	UG15F185P004	UG16F142P002	1
UG15F047P004	UG15F291P001	UG16F151P001	1
UG15F196P020	UG15F168P001	UG16F152P001	1
UG15F047P004	UG15F233P011	UG16F155P003	1
UG15F185P004	UG15F235P007	UG16F174P002	1
UG15F088P003	unknown	UG16F183P015	1
UG15F088P003	unknown	UG16F183P041	1
UG15F088P006	UG15F117P009	UG16F184P006	1
UG15F088P006	UG15F117P009	UG16F184P008	1
UG15F271P008	UG15F087P505	UG16F186P006	1
UG15F008P006	UG15F117P001	UG16F190P007	1
UG15F087P001	UG15F168P009	UG16F191P002	1
UG15F087P016	UG15F271P008	UG16F192P001	1
UG15F087P016	UG15F271P008	UG16F192P004	1
UG15F087P505	UG15F087P016	UG16F193P004	1
UG15F088P006	UG15F117P001	UG16F195P005	1
UG15F088P006	UG15F153P001	UG16F199P001	1
UG15F087P016	UG15F291P001	UG16F203P005	1
UG15F276P015	UG15F088P006	UG16F205P001	1
UG15F271P008	UG15F088P006	UG16F207P002	1
UG15F271P008	UG15F088P006	UG16F207P005	1
UG15F087P001	UG15F168P009	UG16F208P001	1

UG15F087P505	UG15F087P016	UG16F211P003	1
UG15F156P518	unknown	UG16F291P001	1
UG15F156P518	unknown	UG16F291P014	1
UG15F271P008	unknown	UG16F292P023	1
UG15F271P008	unknown	UG16F292P230	1
UG15F271P008	unknown	UG16F292P243	1
UG15F088P005	unknown	UG16F293P013	1
UG15F088P005	unknown	UG16F293P027	1
UG15F088P005	unknown	UG16F293P067	1
UG15F088P005	unknown	UG16F293P081	1
UG15F088P005	unknown	UG16F293P092	1
UG15F088P005	unknown	UG16F293P102	1
UG15F088P005	unknown	UG16F293P126	1
UG15F088P005	unknown	UG16F293P137	1
UG15F088P005	unknown	UG16F293P145	1
UG15F088P005	unknown	UG16F293P181	1
UG15F088P005	unknown	UG16F293P183	1
UG15F088P005	unknown	UG16F293P191	1
UG15F088P005	unknown	UG16F293P201	1
UG15F276P015	unknown	UG16F294P003	1
UG15F276P015	unknown	UG16F294P016	1
UG15F276P015	unknown	UG16F294P036	1
UG15F276P015	unknown	UG16F294P081	1
UG15F276P015	unknown	UG16F294P083	1
UG15F276P015	unknown	UG16F294P083	1
UG15F276P015	unknown	UG16F294P086	1
UG15F276P015	unknown	UG16F294P095	1
UG15F185P004	unknown	UG16F295P056	1
UG15F158P009	unknown	UG16F296P008	1
UG15F158P009	unknown	UG16F296P022	1
UG15F158P009	unknown	UG16F296P025	1
UG15F158P009	unknown	UG16F296P211	1
UG15F237P003	unknown	UG16F300P043	1
UG15F237P003	unknown	UG16F300P056	1
UG15F237P003	unknown	UG16F300P062	1
UG15F153P001	unknown	UG16F301P009	1
UG15F153P001	unknown	UG16F301P020	1
UG15F153P001	unknown	UG16F301P023	1
UG15F088P009	unknown	UG16F306P001	1
UG15F116P007	unknown	UG16F307P008	1
UG15F116P007	unknown	UG16F307P021	1
UG15F116P007	unknown	UG16F307P026	1
UG15F235P007	unknown	UG16F308P091	1
UG15F088P002	unknown	UG16F310P003	1
UG15F087P016	unknown	UG16F312P024	1
UG15F087P016	unknown	UG16F312P028	1
UG15F120P003	unknown	UG16F313P004	1

UG15F120P003	unknown	UG16F313P005	1
UG15F120P003	unknown	UG16F313P006	1
UG15F120P003	unknown	UG16F313P013	1
UG15F120P003	unknown	UG16F313P024	1
UG15F120P003	unknown	UG16F313P034	1
UG15F120P003	unknown	UG16F313P044	1
UG15F120P003	unknown	UG16F313P068	1
UG15F120P003	unknown	UG16F313P075	1
UG15F087P001	unknown	UG16F314P034	1
UG15F087P001	unknown	UG16F314P041	1
UG15F087P001	unknown	UG16F314P042	1
UG15F087P001	unknown	UG16F314P045	1
UG15F087P001	unknown	UG16F314P046	1
UG15F168P009	unknown	UG16F318P003	1
UG15F168P009	unknown	UG16F318P004	1
UG15F168P009	unknown	UG16F318P006	1
UG15F168P009	unknown	UG16F318P014	1
UG15F168P009	unknown	UG16F318P024	1
UG15F253P003	unknown	UG16F319P004	1
UG15F253P003	unknown	UG16F319P007	1
UG15F253P003	unknown	UG16F319P017	1
UG15F047P004	unknown	UG16F320P003	1
UG15F047P004	unknown	UG16F320P008	1
UG15F047P004	unknown	UG16F320P012	1
UG15F047P004	unknown	UG16F320P032	1
UG15F196P020	UG15F088P006	UG16F004P001	2
UG15F196P020	UG15F088P006	UG16F004P014	2
UG15F185P004	UG15F306P034	UG16F005P002	2
UG15F185P004	UG15F306P034	UG16F005P014	2
UG15F306P011	UG15F203P005	UG16F039P020	2
UG15F306P011	UG15F047P002	UG16F042P002	2
UG15F117P006	UG15F203P005	UG16F046P007	2
UG15F306P034	UG15F233P011	UG16F052P001	2
UG15F119P001	UG15F168P001	UG16F055P001	2
UG15F119P001	UG15F168P001	UG16F055P002	2
UG15F117P006	UG15F074P001	UG16F056P004	2
UG15F306P011	UG15F185P004	UG16F057P005	2
UG15F306P011	UG15F306P011	UG16F064P002	2
UG15F306P011	UG15F271P006	UG16F070P003	2
UG15F251P006	UG15F203P005	UG16F073P002	2
UG15F117P012	UG15F203P005	UG16F081P004	2
UG15F271P008	UG15F250P006	UG16F086P004	2
UG15F116P007	UG15F271P008	UG16F088P001	2
UG15F258P004	UG15F015P029	UG16F097P001	2
UG15F119P001	UG15F291P001	UG16F112P001	2
UG15F250P006	UG15F258P004	UG16F118P001	2
UG15F236P003	UG15F096P002	UG16F119P001	2

UG15F118P018	UG15F203P005	UG16F126P001	2
UG15F185P004	UG15F096P004	UG16F134P002	2
UG15F185P004	UG15F258P004	UG16F135P001	2
UG15F196P020	UG15F168P001	UG16F138P005	2
UG15F196P020	UG15F100P004	UG16F140P001	2
UG15F047P004	UG15F306P034	UG16F141P001	2
UG15F271P008	UG15F088P006	UG16F145P002	2
UG15F196P020	UG15F250P006	UG16F147P001	2
UG15F196P020	UG15F250P006	UG16F147P002	2
UG15F196P020	UG15F185P004	UG16F148P001	2
UG15F196P020	UG15F196P007	UG16F150P001	2
UG15F196P020	UG15F196P020	UG16F150P021	2
UG15F185P004	UG15F100P004	UG16F158P005	2
UG15F196P020	UG15F088P006	UG16F162P002	2
UG15F196P020	UG15F116P007	UG16F163P002	2
UG15F196P020	UG15F203P005	UG16F164P006	2
UG15F185P004	UG15F088P006	UG16F168P001	2
UG15F185P004	UG15F276P014	UG16F170P001	2
UG15F185P004	UG15F203P005	UG16F182P001	2
UG15F087P016	UG15F271P008	UG16F185P007	2
UG15F271P008	UG15F087P505	UG16F197P002	2
UG15F271P008	UG15F203P005	UG16F200P001	2
UG15F326P001	UG15F326P001	UG16F201P002	2
UG15F193P010	UG15F235P007	UG16F202P004	2
UG15F236P001	UG15F100P004	UG16F204P003	2
UG15F278P001	UG15F326P001	UG16F209P002	2
UG15F278P001	UG15F326P001	UG16F209P003	2
UG15F196P020	UG15F088P006	UG16F210P001	2
UG15F196P020	UG15F088P006	UG16F210P002	2
UG15F271P008	UG15F087P015	UG16F212P001	2
UG15F116P007	UG15F168P009	UG16F213P002	2
UG15F196P020	unknown	UG16F290P015	2
UG15F196P020	unknown	UG16F290P061	2
UG15F196P020	unknown	UG16F290P093	2
UG15F196P020	unknown	UG16F290P101	2
UG15F196P020	unknown	UG16F290P144	2
UG15F196P020	unknown	UG16F290P181	2
UG15F196P020	unknown	UG16F290P191	2
UG15F196P020	unknown	UG16F290P211	2
UG15F196P020	unknown	UG16F290P218	2
UG15F196P020	unknown	UG16F290P236	2
UG15F196P020	unknown	UG16F290P238	2
UG15F196P020	unknown	UG16F290P284	2
UG15F196P020	unknown	UG16F290P286	2
UG15F196P020	unknown	UG16F290P294	2
UG15F196P020	unknown	UG16F290P294	2
UG15F156P518	unknown	UG16F291P018	2

UG15F156P518	unknown	UG16F291P058	2
UG15F156P518	unknown	UG16F291P071	2
UG15F156P518	unknown	UG16F291P122	2
UG15F156P518	unknown	UG16F291P132	2
UG15F156P518	unknown	UG16F291P139	2
UG15F156P518	unknown	UG16F291P143	2
UG15F156P518	unknown	UG16F291P187	2
UG15F156P518	unknown	UG16F291P222	2
UG15F271P008	unknown	UG16F292P003	2
UG15F271P008	unknown	UG16F292P011	2
UG15F271P008	unknown	UG16F292P045	2
UG15F271P008	unknown	UG16F292P208	2
UG15F271P008	unknown	UG16F292P254	2
UG15F185P004	unknown	UG16F295P005	2
UG15F185P004	unknown	UG16F295P041	2
UG15F185P004	unknown	UG16F295P047	2
UG15F185P004	unknown	UG16F295P063	2
UG15F185P004	unknown	UG16F295P065	2
UG15F306P034	unknown	UG16F297P004	2
UG15F117P006	unknown	UG16F299P005	2
UG15F088P002	unknown	UG16F310P004	2
UG15F088P002	unknown	UG16F310P009	2
UG15F087P016	unknown	UG16F312P023	2
UG15F087P016	unknown	UG16F312P026	2
UG15F276P001	unknown	UG16F316P004	2
UG15F276P001	unknown	UG16F316P012	2
UG15F276P001	unknown	UG16F316P023	2
UG15F168P009	unknown	UG16F318P013	2
UG15F168P009	unknown	UG16F318P016	2
UG15F253P003	unknown	UG16F319P002	2
UG15F253P003	unknown	UG16F319P011	2
UG15F253P003	unknown	UG16F319P026	2
UG15F253P003	unknown	UG16F319P029	2
UG15F253P003	unknown	UG16F319P032	2
UG15F047P004	unknown	UG16F320P002	2
UG15F047P004	unknown	UG16F320P017	2
UG15F047P004	unknown	UG16F320P019	2
UG15F271P008	UG15F233P011	UG16F001P002	3
UG15F271P008	UG15F233P011	UG16F001P006	3
UG15F271P008	UG15F233P011	UG16F001P014	3
UG15F116P007	UG15F233P011	UG16F003P002	3
UG15F236P006	UG15F100P004	UG16F043P001	3
UG15F306P034	UG15F117P012	UG16F050P002	3
UG15F088P006	UG15F235P007	UG16F051P002	3
UG15F088P003	UG15F235P007	UG16F068P001	3
UG15F100P004	UG15F100P004	UG16F080P001	3
UG15F117P009	UG15F233P011	UG16F082P001	3

UG15F117P009	UG15F233P011	UG16F082P002	3
UG15F117P009	UG15F233P011	UG16F082P003	3
UG15F088P006	UG15F253P003	UG16F089P002	3
UG15F168P001	UG15F088P006	UG16F090P002	3
UG15F258P004	UG15F271P008	UG16F091P002	3
UG15F088P003	UG15F235P011	UG16F103P002	3
UG15F117P012	UG15F117P012	UG16F105P002	3
UG15F236P006	UG15F100P004	UG16F106P001	3
UG15F116P007	UG15F235P007	UG16F108P001	3
UG15F117P012	UG15F235P007	UG16F110P001	3
UG15F088P005	UG15F119P001	UG16F113P001	3
UG15F118P018	UG15F235P007	UG16F125P001	3
UG15F235P007	UG15F096P002	UG16F133P004	3
UG15F235P007	UG15F096P002	UG16F133P021	3
UG15F235P007	UG15F203P005	UG16F156P004	3
UG15F235P007	UG15F088P006	UG16F160P005	3
UG15F235P007	UG15F119P001	UG16F161P004	3
UG15F185P004	UG15F271P008	UG16F165P001	3
UG15F185P004	UG15F235P007	UG16F172P012	3
UG15F235P007	UG15F088P003	UG16F175P002	3
UG15F088P003	unknown	UG16F183P032	3
UG15F087P505	UG15F235P007	UG16F194P001	3
UG15F235P007	UG15F120P003	UG16F196P002	3
UG15F233P011	UG15F087P016	UG16F198P002	3
UG15F193P010	UG15F235P007	UG16F202P002	3
UG15F271P008	unknown	UG16F292P078	3
UG15F237P003	unknown	UG16F300P009	3
UG15F237P003	unknown	UG16F300P026	3
UG15F237P003	unknown	UG16F300P041	3
UG15F237P003	unknown	UG16F300P042	3
UG15F237P003	unknown	UG16F300P046	3
UG15F237P003	unknown	UG16F300P053	3
UG15F237P003	unknown	UG16F300P066	3
UG15F100P004	unknown	UG16F302P002	3
UG15F100P004	unknown	UG16F302P021	3
UG15F233P011	unknown	UG16F304P005	3
UG15F233P011	unknown	UG16F304P019	3
UG15F233P011	unknown	UG16F304P025	3
UG15F233P011	unknown	UG16F304P032	3
UG15F233P011	unknown	UG16F304P037	3
UG15F233P011	unknown	UG16F304P052	3
UG15F233P011	unknown	UG16F304P061	3
UG15F233P011	unknown	UG16F304P092	3
UG15F233P011	unknown	UG16F304P165	3
UG15F235P007	unknown	UG16F305P024	3
UG15F235P007	unknown	UG16F308P021	3
UG15F235P007	unknown	UG16F308P041	3

UG15F235P007	unknown	UG16F308P055	3
UG15F235P007	unknown	UG16F308P059	3
UG15F235P007	unknown	UG16F308P079	3
UG15F233P001	unknown	UG16F311P013	3
UG15F087P016	unknown	UG16F312P031	3
UG15F120P003	unknown	UG16F313P033	3
UG15F120P003	unknown	UG16F313P061	3
UG15F120P003	unknown	UG16F313P062	3
UG15F237P007	unknown	UG16F315P001	3
UG15F237P007	unknown	UG16F315P005	3
UG15F237P007	unknown	UG16F315P022	3
UG15F237P007	unknown	UG16F315P032	3
