# Supplementary material for: Identification of Genomic Regions for Traits Associated with Flowering in Cassava (Manihot esculenta Crantz)
Source: Plants (Basel). 2024 Mar 12;13(6):796. doi: 10.3390/plants13060796 (PMC10974989; doi:10.3390/plants13060796)
Supplement: Supplementary file 1 [file plants-13-00796-s001.zip › Plants_Supplementaries2/Supplementary Table 2.pdf]

Supplementary Table 2: Marker-trait associations for flowering-associated traits of 293 cassava accessions

Trait	No. of MTA	Chromosome	Associated SNP marker ID
Branching	15	5 (01)	S5_29309724
		8 (02)	S8_38134897; S8_39184412
		11 (01)	S11_32333764
		15 (01)	S15_11747301
		16 (03)	S16_28288554; S16_28711444; S16_29508150
		18 (07)	S18_263746; S18_597957; S18_1002380; S18_1489472; S18_1562744; S18_1832353; S18_2456168
Branch type	08	5 (01)	S5_29309724
		8 (01)	S8_39184412
		14 (01)	S14_1230231
		15 (01)	S15_11747301
		18 (04)	S18_1002380; S18_1489472; S18_1562744; S18_1832353
Branching levels	14	5 (01)	S5_29309724
		8 (02)	S8_38134897; S8_39184412
		9 (02)	S9_31290323; S9_31489987
		11 (01)	S11_3127381
		14 (01)	S14_1230231
		15 (01)	S15_11747301
		18 (06)	S18_597957; S18_1002380; S18_1489472; S18_1562744; S18_1832353; S18_2456168
Nodes at 1st branch	16	3 (01)	S3_21330906
		5 (02)	S5_22566689; S5_29309724
		8 (01)	S8_39184412
		9 (01)	S9_8276994
		11 (01)	S11_3127381
		14 (06)	S14_964612; S14_1209616; S14_1230231; S14_1653289; S14_2247549; S14_3759369
		15 (01)	S15_11747301
		18 (03)	S18_1002380; S18_1562744; S18_1832353
Stem height at 1st branch	-	-	-

MTA = marker-trait association, number in brackets shows number of MTA on the chromosome
