# Supplementary material for: Identification of Genomic Regions for Traits Associated with Flowering in Cassava (Manihot esculenta Crantz)
Source: Plants (Basel). 2024 Mar 12;13(6):796. doi: 10.3390/plants13060796 (PMC10974989; doi:10.3390/plants13060796)
Supplement: Supplementary file 1 [file plants-13-00796-s001.zip › Plants_Supplementaries2/Supplementary Table 3.pdf]

Supplementary Table 3: Marker-Trait-Associations for Namulonge, Serere and combined data

Data	Trait	SNP	Chrom	Position	P.value	maf	Rsquare without SNP	Rsquare with SNP	effect	PVE (%)
Namulonge	Branch	S5_29309724	5	29309724	1.78E-07	0.01049	0.034124296	0.133081344	4.50678	25.73266104
Namulonge	Branch	S8_39184412	8	39184412	1.64E-07	0.024476	0.034124296	0.133706606	-3.13219	3.39081112
Namulonge	Branch	S18_597957	18	597957	1.97E-08	0.024476	0.034124296	0.149454668	-3.29212	2.093347497
Namulonge	Branch	S18_1002380	18	1002380	1.41E-08	0.024476	0.034124296	0.15196802	-3.34896	2.549851553
Namulonge	Branch	S18_1489472	18	1489472	5.96E-07	0.027972	0.034124296	0.12423185	-2.74205	0
Namulonge	Branch	S18_1562744	18	1562744	1.08E-09	0.020979	0.034124296	0.171571112	-3.90107	4.160323009
Namulonge	Branch	S18_1832353	18	1832353	1.16E-07	0.017483	0.034124296	0.136244556	-3.64257	2.427278381
Serere	Branch	S3_21330906	3	21330906	2.42E-08	0.017986	0.090089415	0.200648371	2.782546	10.7423347
Serere	Branch	S3_25142240	3	25142240	6.17E-07	0.039568	0.090089415	0.177345207	1.711481	4.97448681
Serere	Branch	S8_38134897	8	38134897	5.38E-08	0.05036	0.090089415	0.194827581	-1.91966	1.742343765
Serere	Branch	S8_39184412	8	39184412	1.11E-09	0.02518	0.090089415	0.22345769	-3.08439	0.385317179
Serere	Branch	S11_3127381	11	3127381	5.64E-07	0.007194	0.090089415	0.177985152	4.869953	2.31E-08
Serere	Branch	S11_32333764	11	32333764	6.92E-08	0.017986	0.090089415	0.193001439	3.233244	2.38932066
Serere	Branch	S16_28288554	16	28288554	4.80E-07	0.017986	0.090089415	0.179130437	-2.9924	0.226962201
Serere	Branch	S16_28711444	16	28711444	4.80E-07	0.017986	0.090089415	0.179130437	-2.9924	0.000687306
Serere	Branch	S16_29508150	16	29508150	4.80E-07	0.017986	0.090089415	0.179130437	-2.9924	0.046195133
Serere	Branch	S18_263746	18	263746	4.86E-09	0.05036	0.090089415	0.212430114	-2.02129	1.183811126
Serere	Branch	S18_491437	18	491437	8.93E-07	0.093525	0.090089415	0.174731051	-1.31373	1.64E-07
Serere	Branch	S18_597957	18	597957	1.29E-09	0.032374	0.090089415	0.222301473	-2.65432	0
Serere	Branch	S18_813314	18	813314	1.81E-06	0.071942	0.090089415	0.169750421	-1.53369	0
Serere	Branch	S18_1002380	18	1002380	4.16E-12	0.02518	0.090089415	0.26630294	-3.49152	1.025327568
Serere	Branch	S18_1312039	18	1312039	1.75E-06	0.100719	0.090089415	0.169994607	-1.26389	6.19E-10
Serere	Branch	S18_1489472	18	1489472	3.73E-11	0.02518	0.090089415	0.249226161	-3.23686	4.73E-09
Serere	Branch	S18_1562744	18	1562744	7.90E-17	0.017986	0.090089415	0.355381749	-5.08877	22.12003246
Serere	Branch	S18_1832353	18	1832353	7.13E-10	0.014388	0.090089415	0.226758567	-3.97799	3.67E-09
Serere	Branch	S18_2456168	18	2456168	2.63E-07	0.068345	0.090089415	0.183409236	-1.81993	2.251915378
CombineData	Branch	S5_29309724	5	29309724	1.12E-07	0.010239	0.041285936	0.140274984	1.240582	17.93010207
CombineData	Branch	S8_38134897	8	38134897	1.53E-06	0.047782	0.041285936	0.121831802	-0.58488	0.398989652
CombineData	Branch	S8_39184412	8	39184412	1.26E-09	0.023891	0.041285936	0.172970921	-1.06713	1.182168246

CombineData	Branch	S11_32333764	11	32333764	5.92E-08	0.017065	0.041285936	0.144874059	1.113705	2.47859136
CombineData	Branch	S15_11747301	15	11747301	3.59E-07	0.013652	0.041285936	0.132026951	-1.04514	7.194914177
CombineData	Branch	S16_28288554	16	28288554	4.68E-07	0.017065	0.041285936	0.130147541	-1.03181	1.822947236
CombineData	Branch	S16_28711444	16	28711444	4.68E-07	0.017065	0.041285936	0.130147541	-1.03181	1.34E-06
CombineData	Branch	S16_29508150	16	29508150	4.68E-07	0.017065	0.041285936	0.130147541	-1.03181	0.299617592
CombineData	Branch	S18_263746	18	263746	2.61E-07	0.054608	0.041285936	0.134279564	-0.58732	0.8945882
CombineData	Branch	S18_597957	18	597957	1.07E-08	0.030717	0.041285936	0.157231074	-0.86418	0
CombineData	Branch	S18_1002380	18	1002380	1.28E-11	0.023891	0.041285936	0.207678482	-1.17444	1.27E-08
CombineData	Branch	S18_1489472	18	1489472	2.30E-09	0.027304	0.041285936	0.168557531	-0.94645	7.48E-09
CombineData	Branch	S18_1562744	18	1562744	2.05E-13	0.020478	0.041285936	0.24001633	-1.38013	2.2808549
CombineData	Branch	S18_1832353	18	1832353	3.38E-09	0.017065	0.041285936	0.165711636	-1.17439	1.800534468
CombineData	Branch	S18_2456168	18	2456168	8.58E-07	0.068259	0.041285936	0.125880352	-0.592	0.075619548
Namulonge	Branch1_No	S5_29309724	5	29309724	3.82E-12	0.01049	0.026051672	0.209487475	1.394817	43.70949906
Namulonge	Branch1_No	S13_31910330	13	31910330	5.15E-07	0.020979	0.026051672	0.117977278	0.756149	9.280509192
Namulonge	Branch1_No	S18_597957	18	597957	2.24E-06	0.024476	0.026051672	0.107232305	-0.6086	0.646531749
Namulonge	Branch1_No	S18_1002380	18	1002380	1.89E-06	0.024476	0.026051672	0.108482857	-0.61366	1.279112261
Namulonge	Branch1_No	S18_1562744	18	1562744	3.04E-07	0.020979	0.026051672	0.121866931	-0.71473	0
Namulonge	Branch1_No	S18_1832353	18	1832353	1.43E-08	0.017483	0.026051672	0.144772188	-0.86524	8.375812693
Serere	Branch1_No	S2_16602674	2	16602674	9.32E-08	0.028777	0.027245342	0.134973712	2.052093	2.573737949
Serere	Branch1_No	S3_21330906	3	21330906	5.12E-12	0.017986	0.027245342	0.213887519	3.342325	1.548880719
Serere	Branch1_No	S3_25142240	3	25142240	1.64E-06	0.039568	0.027245342	0.113159204	1.543766	0.402337992
Serere	Branch1_No	S4_15391671	4	15391671	1.22E-07	0.028777	0.027245342	0.132867635	2.072178	18.0009341
Serere	Branch1_No	S4_18758385	4	18758385	1.03E-09	0.021583	0.027245342	0.170438063	2.757781	34.31234855
Serere	Branch1_No	S8_37096672	8	37096672	1.48E-06	0.115108	0.027245342	0.113929062	-1.02101	0.154676572
Serere	Branch1_No	S8_39184412	8	39184412	1.23E-08	0.02518	0.027245342	0.150749152	-2.34841	0.293003774
Serere	Branch1_No	S11_3127381	11	3127381	3.96E-08	0.007194	0.027245342	0.141593923	4.135148	0.999084194
Serere	Branch1_No	S13_3899536	13	3899536	5.02E-08	0.007194	0.027245342	0.139756646	4.062403	0.001496864
Serere	Branch1_No	S13_3929670	13	3929670	5.02E-08	0.007194	0.027245342	0.139756646	4.062403	4.303582973
Serere	Branch1_No	S14_1230231	14	1230231	3.56E-07	0.021583	0.027245342	0.124707166	-2.57062	0
Serere	Branch1_No	S18_597957	18	597957	9.66E-07	0.032374	0.027245342	0.117143367	-1.73888	2.54E-08
Serere	Branch1_No	S18_1002380	18	1002380	1.94E-08	0.02518	0.027245342	0.147158573	-2.27965	0
Serere	Branch1_No	S18_1489472	18	1489472	1.75E-08	0.02518	0.027245342	0.147948557	-2.31371	7.86E-09

Serere	Branch1_No	S18_1562744	18	1562744	2.26E-11	0.017986	0.027245342	0.201511303	-3.26149	0.004287925
Serere	Branch1_No	S18_1832353	18	1832353	5.29E-14	0.014388	0.027245342	0.252897055	-4.09871	22.52931446
CombineData	Branch1_No	S5_29309724	5	29309724	3.22E-10	0.010239	0.011304348	0.157640386	1.648382	36.88183398
CombineData	Branch1_No	S8_39184412	8	39184412	5.62E-08	0.023891	0.011304348	0.118509214	-0.93588	0.105452661
CombineData	Branch1_No	S14_1230231	14	1230231	2.66E-07	0.020478	0.011304348	0.107076025	-1.0957	2.356147613
CombineData	Branch1_No	S15_11747301	15	11747301	1.80E-07	0.013652	0.011304348	0.109931661	-1.17217	12.13825661
CombineData	Branch1_No	S18_1002380	18	1002380	4.14E-08	0.023891	0.011304348	0.120778426	-0.93588	0.157317954
CombineData	Branch1_No	S18_1489472	18	1489472	7.06E-07	0.027304	0.011304348	0.099951815	-0.79392	0
CombineData	Branch1_No	S18_1562744	18	1562744	3.23E-09	0.020478	0.011304348	0.139967519	-1.09604	0
CombineData	Branch1_No	S18_1832353	18	1832353	5.62E-11	0.017065	0.011304348	0.171239545	-1.32908	11.21675484
Namulonge	Branch_Levels	S2_27713636	2	27713636	2.92E-07	0.026224	0.024810333	0.121063793	-0.73808	2.596455472
Namulonge	Branch_Levels	S5_29309724	5	29309724	3.36E-16	0.01049	0.024810333	0.287212589	1.72939	44.91028335
Namulonge	Branch_Levels	S8_39184412	8	39184412	5.01E-10	0.024476	0.024810333	0.169564944	-0.92904	1.417852581
Namulonge	Branch_Levels	S9_31489987	9	31489987	5.48E-07	0.052448	0.024810333	0.116404878	-0.52105	0.92716512
Namulonge	Branch_Levels	S15_11747301	15	11747301	2.24E-07	0.013986	0.024810333	0.12301745	-0.93094	9.504693402
Namulonge	Branch_Levels	S18_597957	18	597957	2.99E-11	0.024476	0.024810333	0.191865371	-0.96541	0
Namulonge	Branch_Levels	S18_813314	18	813314	4.23E-07	0.062937	0.024810333	0.118306158	-0.52454	0.157051549
Namulonge	Branch_Levels	S18_1002380	18	1002380	1.81E-11	0.024476	0.024810333	0.195908157	-0.98883	2.871798446
Namulonge	Branch_Levels	S18_1489472	18	1489472	1.56E-09	0.027972	0.024810333	0.160694072	-0.81821	0
Namulonge	Branch_Levels	S18_1562744	18	1562744	2.86E-13	0.020979	0.024810333	0.229763908	-1.16551	6.25E-08
Namulonge	Branch_Levels	S18_1832353	18	1832353	2.33E-10	0.017483	0.024810333	0.175564573	-1.07842	1.81037709
Namulonge	Branch_Levels	S18_2456168	18	2456168	5.36E-07	0.06993	0.024810333	0.116567214	-0.51738	2.74E-07
Serere	Branch_Levels	S3_21330906	3	21330906	8.88E-10	0.017986	0.028147694	0.172362702	0.555311	7.727143488
Serere	Branch_Levels	S4_18758385	4	18758385	8.35E-08	0.021583	0.028147694	0.136620351	0.453587	9.290335859
Serere	Branch_Levels	S8_38134897	8	38134897	2.58E-07	0.05036	0.028147694	0.12796063	-0.28101	3.4441899
Serere	Branch_Levels	S8_39184412	8	39184412	2.57E-07	0.02518	0.028147694	0.127993272	-0.39637	0
Serere	Branch_Levels	S11_3127381	11	3127381	6.61E-07	0.007194	0.028147694	0.1208227	0.698069	0
Serere	Branch_Levels	S13_3899536	13	3899536	1.49E-06	0.007194	0.028147694	0.114680572	0.672835	3.2845138
Serere	Branch_Levels	S13_3929670	13	3929670	1.49E-06	0.007194	0.028147694	0.114680572	0.672835	13.1497843
Serere	Branch_Levels	S18_1002380	18	1002380	4.60E-07	0.02518	0.028147694	0.123576602	-0.38222	2.00E-09
Serere	Branch_Levels	S18_1489472	18	1489472	3.58E-07	0.02518	0.028147694	0.125476527	-0.39071	6.20E-09
Serere	Branch_Levels	S18_1562744	18	1562744	1.60E-09	0.017986	0.028147694	0.167644673	-0.54813	0.663631965

Serere	Branch_Levels	S18_1832353	18	1832353	9.47E-12	0.014388	0.028147694	0.209483726	-0.69067	19.58646122
CombineData	Branch_Levels	S5_29309724	5	29309724	9.70E-12	0.010239	0.019129801	0.191572807	1.537258	31.64090939
CombineData	Branch_Levels	S8_38134897	8	38134897	1.58E-07	0.047782	0.019129801	0.117911124	-0.59523	0.815391937
CombineData	Branch_Levels	S8_39184412	8	39184412	1.44E-10	0.023891	0.019129801	0.170528825	-1.0506	0.193603102
CombineData	Branch_Levels	S9_31290323	9	31290323	2.18E-06	0.03413	0.019129801	0.09900238	-0.67435	0
CombineData	Branch_Levels	S9_31489987	9	31489987	2.23E-06	0.051195	0.019129801	0.098844431	-0.53878	0.308272181
CombineData	Branch_Levels	S11_3127381	11	3127381	2.15E-06	0.010239	0.019129801	0.099102552	1.13153	1.067507616
CombineData	Branch_Levels	S14_1230231	14	1230231	4.25E-07	0.020478	0.019129801	0.110732072	-1.00069	1.930112918
CombineData	Branch_Levels	S15_11747301	15	11747301	4.64E-09	0.013652	0.019129801	0.144043522	-1.15038	11.32883332
CombineData	Branch_Levels	S18_597957	18	597957	6.30E-09	0.030717	0.019129801	0.14173972	-0.81636	0.102286928
CombineData	Branch_Levels	S18_1002380	18	1002380	7.79E-12	0.023891	0.019129801	0.193305851	-1.10345	1.647890066
CombineData	Branch_Levels	S18_1489472	18	1489472	7.03E-10	0.027304	0.019129801	0.158331009	-0.91394	0
CombineData	Branch_Levels	S18_1562744	18	1562744	7.93E-14	0.020478	0.019129801	0.23020971	-1.30755	0
CombineData	Branch_Levels	S18_1832353	18	1832353	6.39E-12	0.017065	0.019129801	0.194872308	-1.28583	4.841044264
CombineData	Branch_Levels	S18_2456168	18	2456168	3.28E-07	0.068259	0.019129801	0.112618878	-0.57597	0.111587424
Namulonge	Branch1_Nodes	S2_15333250	2	15333250	1.70E-06	0.027972	0.011494807	0.095914665	-1.5865	0.54332561
Namulonge	Branch1_Nodes	S2_27713636	2	27713636	5.49E-08	0.026224	0.011494807	0.121696771	-1.98425	6.824215668
Namulonge	Branch1_Nodes	S5_29309724	5	29309724	2.21E-11	0.01049	0.011494807	0.183287034	3.705906	33.37429407
Namulonge	Branch1_Nodes	S8_39184412	8	39184412	5.65E-07	0.024476	0.011494807	0.104112011	-1.80455	2.25E-07
Namulonge	Branch1_Nodes	S9_8276994	9	8276994	1.74E-07	0.038462	0.011494807	0.112952828	1.641282	0.70901025
Namulonge	Branch1_Nodes	S12_9117572	12	9117572	2.65E-09	0.017483	0.011494807	0.145094243	-2.52579	13.14257273
Namulonge	Branch1_Nodes	S14_1230231	14	1230231	6.34E-07	0.020979	0.011494807	0.103252312	-2.22504	1.356125483
Namulonge	Branch1_Nodes	S15_11747301	15	11747301	2.02E-11	0.013986	0.011494807	0.184002066	-3.21623	12.96773119
Namulonge	Branch1_Nodes	S18_597957	18	597957	3.44E-07	0.024476	0.011494807	0.107819456	-1.82009	2.72E-07
Namulonge	Branch1_Nodes	S18_1002380	18	1002380	2.86E-07	0.024476	0.011494807	0.109206412	-1.83431	6.66E-05
Namulonge	Branch1_Nodes	S18_1562744	18	1562744	3.51E-08	0.020979	0.011494807	0.125117617	-2.13685	3.13E-07
Namulonge	Branch1_Nodes	S18_1832353	18	1832353	1.50E-09	0.017483	0.011494807	0.149546247	-2.56162	2.507672965
Serere	Branch1_Nodes	S1_24720107	1	24720107	6.48E-07	0.010791	0.032995319	0.125362661	-2.57412	3.526363745
Serere	Branch1_Nodes	S2_16602674	2	16602674	4.80E-10	0.028777	0.032995319	0.181409882	2.067939	3.144503806
Serere	Branch1_Nodes	S3_21330906	3	21330906	3.37E-15	0.017986	0.032995319	0.281332345	3.313648	6.3779189
Serere	Branch1_Nodes	S3_25142240	3	25142240	5.17E-08	0.039568	0.032995319	0.144613887	1.509473	0.202692414
Serere	Branch1_Nodes	S4_6193028	4	6193028	6.84E-07	0.010791	0.032995319	0.124950203	2.58666	2.450684184

Serere	Branch1_Nodes	S4_15391671	4	15391671	8.05E-10	0.028777	0.032995319	0.177269599	2.079253	6.112979435
Serere	Branch1_Nodes	S4_18758385	4	18758385	2.28E-12	0.021583	0.032995319	0.225306934	2.745775	19.07339948
Serere	Branch1_Nodes	S5_22566689	5	22566689	7.31E-08	0.010791	0.032995319	0.141943704	-2.77254	4.329653592
Serere	Branch1_Nodes	S10_26312279	10	26312279	3.70E-07	0.010791	0.032995319	0.129583508	2.627104	3.491960521
Serere	Branch1_Nodes	S11_3127381	11	3127381	8.53E-11	0.007194	0.032995319	0.195373142	4.175304	12.94470182
Serere	Branch1_Nodes	S13_3899536	13	3899536	5.00E-10	0.007194	0.032995319	0.181092882	3.999875	4.736657495
Serere	Branch1_Nodes	S13_3926842	13	3926842	7.01E-07	0.010791	0.032995319	0.124764065	2.590522	5.85E-07
Serere	Branch1_Nodes	S13_3929670	13	3929670	5.00E-10	0.007194	0.032995319	0.181092882	3.999875	4.132706196
Serere	Branch1_Nodes	S14_964612	14	964612	9.58E-08	0.010791	0.032995319	0.139871951	-2.77925	5.47E-08
Serere	Branch1_Nodes	S14_1209616	14	1209616	9.58E-08	0.010791	0.032995319	0.139871951	-2.77925	9.72E-08
Serere	Branch1_Nodes	S14_1230231	14	1230231	3.43E-10	0.021583	0.032995319	0.18411978	-2.72276	4.04E-08
Serere	Branch1_Nodes	S14_1653289	14	1653289	9.58E-08	0.010791	0.032995319	0.139871951	-2.77925	2.83E-07
Serere	Branch1_Nodes	S14_2247549	14	2247549	9.58E-08	0.010791	0.032995319	0.139871951	-2.77925	0
Serere	Branch1_Nodes	S14_3759369	14	3759369	9.58E-08	0.010791	0.032995319	0.139871951	-2.77925	3.27E-08
Serere	Branch1_Nodes	S15_11747301	15	11747301	2.10E-06	0.014388	0.032995319	0.116550407	-2.13686	1.73669633
Serere	Branch1_Nodes	S16_3062172	16	3062172	2.22E-06	0.014388	0.032995319	0.116140593	-2.11112	1.584945026
Serere	Branch1_Nodes	S16_27842042	16	27842042	2.10E-07	0.010791	0.032995319	0.133891552	-2.66842	3.313827688
Serere	Branch1_Nodes	S18_9295397	18	9295397	3.20E-07	0.079137	0.032995319	0.1306885	-1.1186	0
CombineData	Branch1_Nodes	S3_21330906	3	21330906	1.45E-07	0.017065	0.005607584	0.106371161	0.81666	12.93879494
CombineData	Branch1_Nodes	S5_22566689	5	22566689	5.75E-08	0.010239	0.005607584	0.11326397	-1.06798	4.845146622
CombineData	Branch1_Nodes	S5_29309724	5	29309724	5.16E-07	0.010239	0.005607584	0.097060368	0.994939	20.98497522
CombineData	Branch1_Nodes	S8_39184412	8	39184412	6.61E-07	0.023891	0.005607584	0.095254701	-0.65689	0
CombineData	Branch1_Nodes	S9_8276994	9	8276994	5.00E-07	0.037543	0.005607584	0.097288611	0.574125	0.267967021
CombineData	Branch1_Nodes	S11_3127381	11	3127381	1.23E-07	0.010239	0.005607584	0.107640226	1.051692	2.251685411
CombineData	Branch1_Nodes	S14_964612	14	964612	1.53E-07	0.010239	0.005607584	0.106014049	-1.04697	0.084190779
CombineData	Branch1_Nodes	S14_1209616	14	1209616	1.53E-07	0.010239	0.005607584	0.106014049	-1.04697	0.01209467
CombineData	Branch1_Nodes	S14_1230231	14	1230231	7.73E-11	0.020478	0.005607584	0.163951908	-1.08049	1.606297091
CombineData	Branch1_Nodes	S14_1653289	14	1653289	1.53E-07	0.010239	0.005607584	0.106014049	-1.04697	0.773141397
CombineData	Branch1_Nodes	S14_2247549	14	2247549	1.53E-07	0.010239	0.005607584	0.106014049	-1.04697	5.27E-07
CombineData	Branch1_Nodes	S14_3759369	14	3759369	1.53E-07	0.010239	0.005607584	0.106014049	-1.04697	0.002412489
CombineData	Branch1_Nodes	S15_11747301	15	11747301	1.65E-11	0.013652	0.005607584	0.176178485	-1.17828	15.23808294
CombineData	Branch1_Nodes	S18_1002380	18	1002380	6.93E-07	0.023891	0.005607584	0.094902853	-0.64814	0

CombineData	Branch1_Nodes	S18_1562744	18	1562744	7.50E-08	0.020478	0.005607584	0.111286995	-0.76154	1.29E-08
CombineData	Branch1_Nodes	S18_1832353	18	1832353	3.01E-09	0.017065	0.005607584	0.135545918	-0.91801	0

Chrom, chromosome; maf, minor allele frequency; PVE, phenotype variance explained. Highlighted rows show MTAs with PVE ≥ 4%, and the rows heighted in green show MTAs common in the two locations. The 'Rsquare with SNP' value is a regression coefficient of the model with SNP, while the 'Rsquare without SNP' value is a regression coefficient of the model without SNP, both contribute to estimation of the PVE.
