# Supplementary material for: Identification of Genomic Regions for Traits Associated with Flowering in Cassava (Manihot esculenta Crantz)
Source: Plants (Basel). 2024 Mar 12;13(6):796. doi: 10.3390/plants13060796 (PMC10974989; doi:10.3390/plants13060796)
Supplement: Supplementary file 1 [file plants-13-00796-s001.zip › Plants_Supplementaries2/Supplementary Table 4.pdf]

Supplementary Table 4: Associated markers and putative candidate genes in the vicinity of 50 Kbp either side for cassava flowering-associated traits

Trait	Associated SNP ID	Chr.	P.value	MAF	R^2	SNP Effect	PVE (%)	Putative candidate gene ID (Phytozome v13; Manihot 7.1)	Gene functional annotation (according to NCBI)
Nodes at 1st branch	S3_21330906 (Chromosome03: 21280836..21380869)	3	1.45 $\times 10^{-7}$	0.017	0.106	0.82	12.94	Manes.03G102000	dol-P-Man:Man(6)GlcNAc(2)-PP-Dol alpha-1,2-mannosyltransferase
								Manes.03G102100	olee1-like protein
								Manes.03G101900	phosphoenolpyruvate carboxylase
								Manes.03G102600	release factor glutamine methyltransferase
								Manes.03G102400	TPR repeat-containing thioredoxin TTL1
								Manes.03G102200	ubiquitin-conjugating enzyme E2 36
								Manes.03G101800	uncharacterized LOC110611895
								Manes.03G102300	uncharacterized LOC110611572
								Manes.03G102500	uncharacterized LOC110611241
	S5_22566689 (Chromosome05: 22516670..22616670)	5	5.75 $\times 10^{-8}$	0.010	0.113	-1.07	4.85	Manes.05G131430	mogroside IE synthase
								Manes.05G131410	mogroside IE synthase-like (LOC122721352), transcript variant X1
								Manes.05G131420	mogroside IE synthase-like (LOC122721259)
								Manes.05G131440	probably inactive leucine-rich repeat receptor-like protein kinase At5g48380
Branch type	S18_1832353 (Chromosome18: 1782341..1882341)	18	5.6 $\times 10^{-11}$	0.017	0.171	-1.33	11.2	Manes.18G016372	protein IQ-DOMAIN 32
Branching levels			6.4 $\times 10^{-12}$	0.017	0.195	1.29	4.84	Manes.18G016200	cytochrome P450 83B1
								Manes.18G016212	protein RCC2
								Manes.18G016400	uncharacterized LOC122722385
								Manes.18G016450	uncharacterized LOC122722384
								Manes.18G016500	histone H3.3
								Manes.18G016700	aldehyde oxidase GLOX
								Manes.18G016725	lysine-specific histone demethylase 1 homolog 3
								Manes.18G016750	V-type proton ATPase 16 kDa proteolipid subunit
								Manes.18G016775	vicilin-like seed storage protein At2g28490
								Manes.18G016800	lysine-specific histone demethylase 1 homolog 3
								Manes.18G017000	V-type proton ATPase 16 kDa proteolipid subunit
Branching	S5_29309724 (Chromosome05:	5	1.12 $\times 10^{-7}$	0.010	0.140	1.24	17.93	Manes.05G186300	reticulon-4-interacting protein 1 homolog, mitochondrial

Branch type	29259752..29359752)		3.21 x10^{-10}	0.010	0.157	1.65	36.88	Manes.05G186500	receptor-like protein kinase 7
Branching levels			9.70 x10^{-12}	0.010	0.191	1.54	31.64	Manes.05G186400	xylulose kinase 2
Nodes at 1st branch			5.16 x10^{-7}	0.010	0.097	0.99	20.98	Manes.05G186600	sucrose transport protein SUC4
								Manes.05G186700	protein DETOXIFICATION 48
Branching	S15_11747301 (Chromosome15: 11697330..11797330)	15	3.6 x10^{-7}	0.014	0.132	-1.05	7.19	Manes.15G140200	uncharacterized LOC110601339
Branch type			1.8 x10^{-7}	0.014	0.110	-1.17	12.14	Manes.15G140300	non-specific lipid-transfer protein 4.1
Branching levels			4.6 x10^{-9}	0.014	0.144	-1.15	11.33	Manes.15G140400	protein NRT1/ PTR FAMILY 8.3
Nodes at 1st branch			1.7 x10^{-11}	0.014	0.176	-1.18	15.24	Manes.15G140500	eukaryotic translation initiation factor 3 subunit I
								Manes.15G140600	short-chain dehydrogenase reductase ATA1
								Manes.15G140700	NADH dehydrogenase [ubiquinone] 1 beta subcomplex subunit 7-like
								Manes.15G140900	myb family transcription factor MOF1
								Manes.15G141000	uncharacterized LOC110600802
								Manes.15G141100	endonuclease V (LOC110601526)
								Manes.15G141200	endonuclease V (LOC110601527)
								Manes.15G141300	protein indeterminate-domain 5, chloroplastic (LOC110600836)
								Manes.15G141400	protein indeterminate-domain 5, chloroplastic (LOC110602325)
								Manes.15G141500	protein CHROMATIN REMODELING 19
								Manes.15G141700	transcription factor bHLH111

Chr., chromosome; MAF, minor allele frequency; PVE, phenotype variance explained; NCBI, National Centre for Biotechnology Information. Number attached by an underscore on each associated SNP represents its physical position on the chromosome. Numbers in brackets below each SNP show interval of gene search, 50 Kbp up or down the physical position of the SNP. The R^2 value presented in this table is a representation of the regression coefficient of the SNP with the model, which partly contributed to estimation of the PVE
